# Effect of Microplastics on the Removal of Nitrogen and Phosphorus from Synthetic Piggery Digestate by Microalgae

**DOI:** 10.3390/polym14204349

**Published:** 2022-10-15

**Authors:** Xiaoai Lin, Longzao Luo, Zhitong Mao, Huimin Wang, Shiyu Chu, Hui Wang, Shuang Luo

**Affiliations:** 1 School of Chemistry and Environmental Science, Shangrao Normal University, Shangrao 334001, China; 2 College of Resources and Environment, Hunan Agricultural University, Changsha 410128, China; 3 College of Eco-Environmental Engineering, Guizhou Minzu University, Guiyang 550003, China

**Keywords:** microalgae, microplastic, nitrogen and phosphorus, synthetic piggery digestate

## Abstract

Microplastics (MPs) have been proven to be one of the major threats to the wastewater treatment system. However, the impact of MPs on microalgae-based wastewater treatment technology are still unknown. In this study, effects of polyvinyl chloride (PVC), polypropylene (PP), and polyethylene (PE) on the removal of nitrogen and phosphorus from synthetic piggery digestate by microalgae were investigated. Results show that the effect of PVC particle size on the growth of microalgae was significant. The effects of PVC content, PP particle size and content, PET particle size and content on the growth of microalgae were all not significant. The removal of ammonia nitrogen by microalgae was influenced significantly by PVC particle size and content, PP particle size and content, the effects of PET particle size and content on the removal of ammonia nitrogen were all not significant. The effects of particle size and content for three MPs on the removal of total phosphorus were all significant. Microalgae cells were bound together after being adsorbed by MPs, which increased the secretion of extracellular polymers and influenced the removal of ammonia nitrogen and total phosphorus.

## 1. Introduction

Cultivation of microalgae in wastewater has been proven a cost-effective technology for wastewater treatment [1]. Microalgae-based wastewater treatment has been studied in various wastewaters, including piggery wastewater [2], domestic wastewater [3], and agro-industrial wastewater [4]. Piggery digestate usually contains high concentrations of ammonia nitrogen (NH_4_-N), total phosphorus (TP), and chemical oxygen demand (COD), which poses a challenge for conventional low-cost treatment processes. Microalgae have the ability to absorb and store nutrients such as carbon (C), nitrogen (N), and phosphorus (P) from wastewater [5]. Moreover, the acquired microalgae biomass contains high contents of hydrocarbon and triacylglycerol, which can be transferred into ethanol and biodiesel, respectively [6]. Therefore, microalgae show great potential for the treatment of piggery digestate.

Recently, microplastics (MPs) widely occur in various environments due to the widespread use of plastics in the agricultural, industrial, and medical fields, among others, and their negative impact on the environment has attracted widespread attention [7]. Sewage-treatment plants have been a major source of microplastics [8]. The presence of MPs will affect wastewater treatment. Green et al. revealed that MPs inhibited the denitrification process, mainly by changing the microbial mediated process of controlling NH_4_^+^ production and reduction, thus leading to the accumulation of ammonia nitrogen in water [9]. Ling et al. found that MPs have a weak negative correlation with the removal process of phosphorus in wastewater and a positive correlation with the biological transformation process of nitrogen [10]. MPs can affect the growth of microalgae, which led to the reduction of chlorophyll content [11], photosynthetic activity, and shading effect [12], thus affecting the nutrient removal by the microalgae. Polyvinyl chloride (PVC), polypropylene (PP), and polyethylene terephthalate (PET) are common MPs in the wastewater of livestock farms [13]. However, the effects of these MPs on the process of nutrient removal by microalgae are still unknown.

In this study, the effects of PVC, PP, and PET with particle size and content on the removal of nitrogen and phosphorus from synthetic piggery digestate by microalgae were investigated. The results of this study are expected to not only provide a theoretical basis and technical guidance for the economical and effective utilization of piggery digestate, but also play an important role in controlling the discharge of MPs from piggery digestate to the environment and ensuring the safety of the ecosystem.

## 2. Materials and Methods

### 2.1. Microalgae and Wastewater

The microalgae *Desmodesmus* sp. CHX1 was obtained from a local oxidation pond of Hangzhou City, China [14]. Microalgae cells were cultured in 250-mL flasks containing 200 mL synthetic piggery digestate, which was prepared based on BG-11 medium. The flasks were kept in illuminating and oscillating incubator at 30 °C and 150 rpm, light intensity of 6000 μmol photons m^−2^ s^−1^, and the daily light/dark cycle of 24 h/0 h. The synthetic piggery digestate was prepared on the basis of the modified BG11 medium, with TP of 45.38 mg/L, NH_4_-N of 192.48 mg/L, pH of 6.85, COD of 560.32 mg/L, respectively.

### 2.2. Experimental Design

The experiment was performed in 250-mL flasks containing 200 mL synthetic wastewater. The initial inoculation of microalgae cells was 0.1 g/L. For the MPs particle size experiments, particle sizes of PVC, PP, and PET were set as 0.15 mm, 0.025 mm, 0.013 mm, and 0.0065 mm, respectively, with MPs content of 70 mg/L. For the MPs content experiments, contents of PVC (0.0065 mm), PP (0.015 mm), and PET (0.013 mm) were set as 10, 40, 70, and 100 mg/L, respectively. Control treatment (CK, without adding MPs) were set up at the same time. Three duplications were set for each treatment. Microalgae biomass, NH_4_-N, and TP concentrations of synthetic wastewater were measured after three days of incubation.

### 2.3. Analysis Methods

The pH value was determined by a pH meter (Leici PHS-3E); NH_4_-N and TP were measured by using Nessler’s reagent colorimetric methods and ammonium molybdate spectrophotometry, respectively. Microalgae biomass was characterized by chlorophyll content, which was determined with spectrophotometer and was calculated according to the method reported by Li et al. [15]. The microstructure of microalgal cells was analyzed by using a scanning electron microscope (SEM, Hitachi SU8000, Tokyo, Japan). The variations of organic matter composition in the synthetic piggery digestate were determined by three-dimensional fluorescence spectroscopy (3D-EEM, HITACHI, F-7000, Tokyo, Japan).

### 2.4. Data Analysis

IBM SPSS version 20.0 software was used for one-way ANOVA analysis in each treatment. The significance level was set to 0.05. Excel (2019) and Origin 2019 software were used for other data analysis and plotting.

## 3. Results and Discussion

### 3.1. Effect of MPs on the Growth of Microalgae

**Figure 1:**
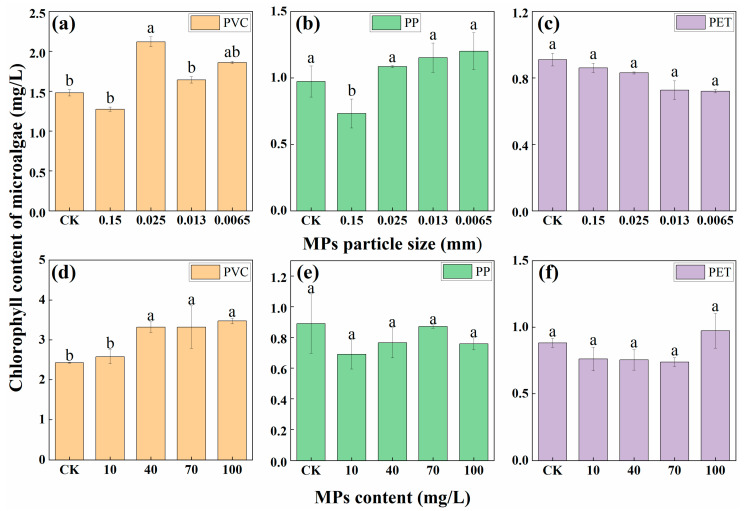
Chlorophyll content of microalgae treated by MPs with different particle sizes (**a**–**c**) and contents (**d**–**f**). Different letters above the error bars indicate statistically significant differences (*p* < 0.05).

The effects of different microplastic particle sizes and contents on the growth of microalgae were shown in Figure 1. Compared with the control treatment, the microalgae biomass of 0.15 mm PVC MPs treatment decreased, whereas other treatments showed an increasing trend, and the most significant increase was observed at 0.025 mm treatment. Microalgae biomass in the 0.15 mm PP MPs treatment showed a decreasing trend, whereas other treatments indicated an upward trend, and the overall effect was not significant. Microalgae biomass in the treatments of PET MPs showed decreasing trends with the decrease of particle size, but the variation trends were not significant. As shown in Figure 1d–f, effects of three MPs content on the growth of microalgae were all not significant. Song et al. reported that 200 mg/L of PET and PVC had the most obvious promoting effect on microalgae (*Chlorella* sp. L38) [16]. Wu et al. found that high concentrations (50–500 mg/L) of PP considerably inhibited the photosynthesis system of two algae (*C. pyrenoidosa* and *M. flos-aquae*) [17].

In this study, the microalgae biomass manifested a downward trend when the particle size of three microplastics—PVC, PP, and PET—were all 0.15 mm. This might be because large particle size MPs can prevent direct light irradiation to microalgae, thereby reducing the photosynthesis of microalgae and inhibiting the growth of microalgae [18]. The three types of MPs had both inhibited and promoted effects on the growth of microalgae. The possible reason for the inhibiting effect was that MPs covered the surface of the algal liquid, which affected the dissolved oxygen content and light intensity. Schwab et al. suggested that the shading effect of particles could reduce algal photosynthesis [19]. Sjollema et al. found that although MPs could block some parts of the light, the rest of the light could still meet the needs of algal cells for photosynthesis [20]. The shading effect was obviously related to the content of MPs. Moreover, small-sized MPs attached on the surface of algal cells would also pose a shading effect, reducing the light absorption efficiency and photosynthesis rate [19]. At the same time, MPs could also exert toxic effects on microalgae, thereby inhibiting the respiration, photosynthesis, and metabolism of microalgae. Moreover, MPs can cause oxidative damage to algal cells, oxidative stress, and intracellular oxidative stress [21]. Meanwhile, the reason for the promoting effect might be that with the increase of the action time for microplastics, microalgae became resistant to MPs. Microalgae adapted to the stress of MPs, improved the physiological properties of algal cells, and could be more effective. To resist external pressure, the photosynthetic activity was enhanced to a certain extent, so that the microalgae could continue its growth and the chlorophyll content was increased slightly under the better growth conditions [22]. Song et al. showed that MPs affected under the concentration of algae was higher than that in the control medium, which suggested that microplastics might end up providing a favorable surrounding for the algae [16].

### 3.2. Effect of MPs on the Removal of NH_4_-N and TP

#### 3.2.1. Removal of NH_4_-N

**Figure 2:**
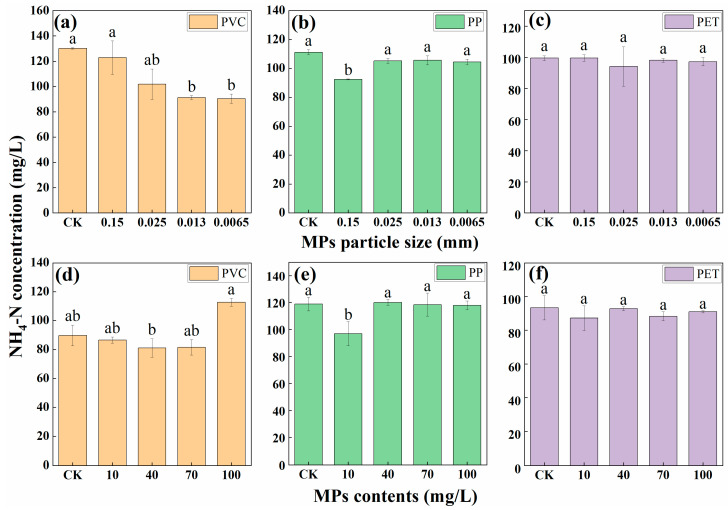
NH_4_-N concentration in wastewater treated by MPs with different particle sizes (**a**–**c**) and contents (**d**–**f**). Different letters above the error bars indicate statistically significant differences (*p* < 0.05).

As shown in Figure 2a, PVC MPs with particle size of 0.15 mm, 0.025 mm, 0.013 mm, and 0.0065 mm can promote the removal of NH_4_-N by microalgae, and the most obvious promotion was observed in the PVC MPs with particle size of 0.013 mm and 0.0065 mm. The removal of NH_4_-N in PP MPs with particle size of 0.15 mm was obviously reduced compared with CK treatment. PP MPs with other particle sizes exert no significant effects on the removal of NH_4_-N by *Desmodesmus* sp. CHX1. The removal efficiencies of NH_4_-N in the treatments of PET MPs with different particle sizes were not significantly different from that of the CK treatment.

Comparing with the CK treatment, the removal of NH_4_-N by PVC MPs with content of 40 mg/L increased by 8.71 mg/L, the removal of NH_4_-N by PVC MPs with content of 100 mg/L reduced by 22.86 mg/L. The removal of NH_4_-N in treatment of PP MPs with content of 10 mg/L was significantly reduced, and no significant differences were observed in treatments of PP MPs with other contents. The removal efficiencies of NH_4_-N in the treatments of PET MPs with different contents were not significantly different from those of the CK treatment. Therefore, MPs most affect the removal of NH_4_-N by *Desmodesmus* sp. CHX1 was PVC MPs, and the effects of PVC MPs on the removal of NH_4_-N become greater with the decreasing of particle size. This was consistent with the study of Zhang et al. [23]. The effects of PET MPs were not as obvious as other two types of MPs.

#### 3.2.2. Removal of TP

**Figure 3:**
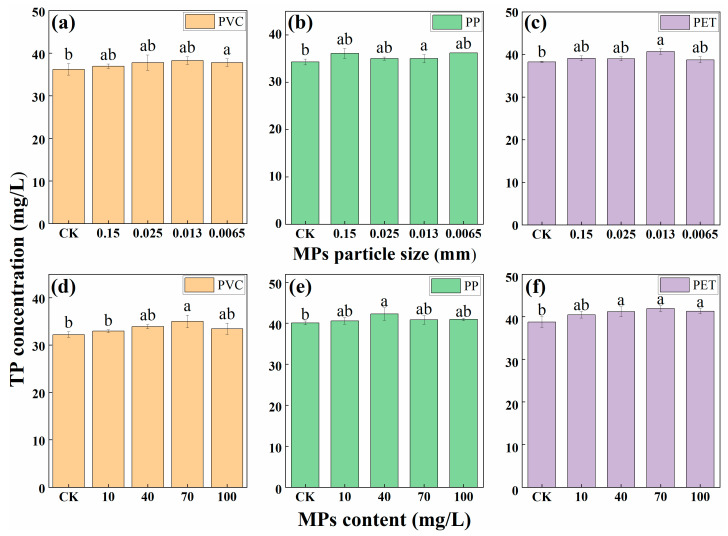
TP concentration in wastewater treated by MPs with different particle sizes (**a**–**c**) and contents (**d**–**f**). Different letters above the error bars indicate statistically significant differences (*p* < 0.05).

The variations of TP concentration under MPs particle sizes and contents were depicted in Figure 3. As shown in Figure 3, TP concentration first increased and then decreased slightly in all treatments compared with the CK treatment after three days of incubation. This indicated that the removal efficiency of TP by *Desmodesmus* sp. CHX1 decreased due to the influence of MPs. The effects of MPs on the removal of TP increase at first and then decrease with the decreasing of particle size. TP concentration first showed an increase and then a decreasing trend with the increase of MPs contents. This meant that the removal efficiency of TP by *Desmodesmus* sp. CHX1 first decreased and then increased due to the influence of MPs with different contents. The possible reason was that microplastics were distributed in the algal fluid and cover the surface of microalgae, which affected the photosynthesis of microalgae and the content of dissolved oxygen in the wastewater. At the same time, microplastics have a toxic effect on microalgae, leading to the decline of some algae, and the decline of algae will release nitrogen and phosphorus, so that the nitrogen and phosphorus in the wastewater could rise again.

### 3.3. Interaction between Microalgae and MPs

**Figure 4:**
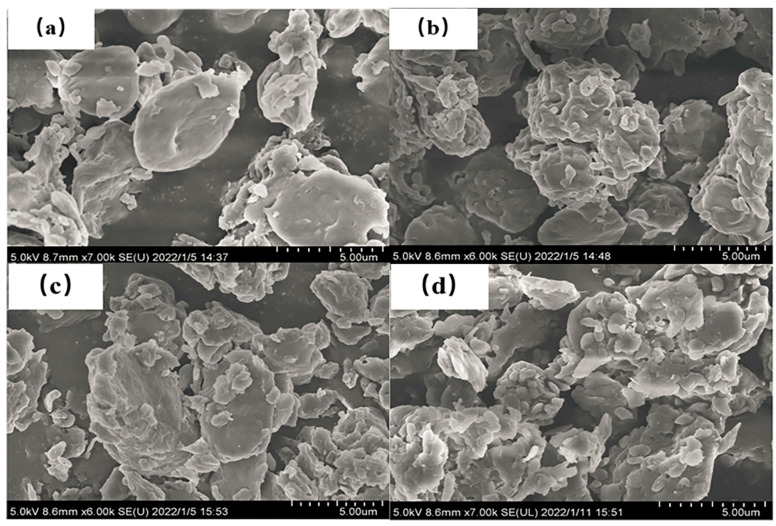
SEM images of microalgae before (**a**) and after treatment by PVC (**b**), PP (**c**), and PET (**d**) MPs.

**Figure 5:**
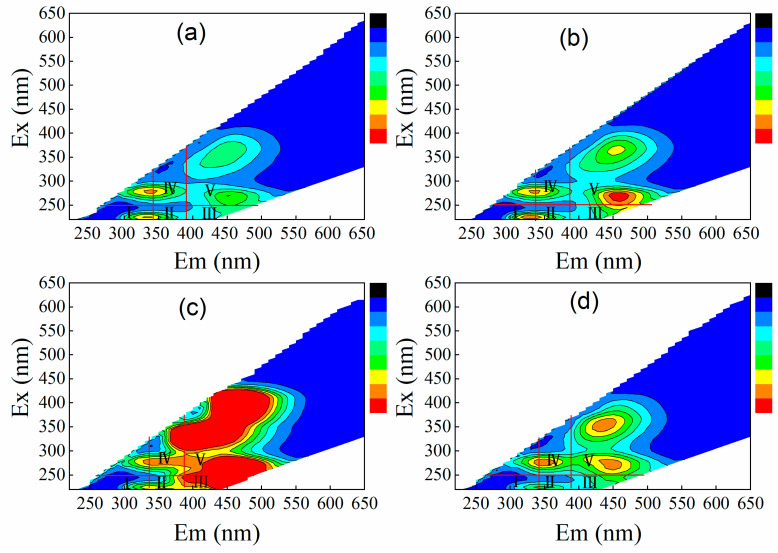
Variations of organic composition in the wastewater before (**a**) and after treatment by PVC (**b**), PP (**c**), and PET (**d**) MPs.

Cell surface structures of microalgae were characterized by SEM (Figure 4). It was observed that the microalgae cells were complete, spherical and dispersed before the treatment by MPs, whereas the microalgae cells became smaller and rough after the treatment by MPs. From the SEM micrograph, it can be seen that microalgae cells were bound together after being adsorbed by microplastics, and the aggregation effect of PET MPs on microalgae cell was more obvious. The interaction between microalgae and MPs is probably caused by hetero-aggregation [18]. Extracellular polymeric substance (EPS) secretion by microalgae could attach to MPs through hydrogen bonds or electrostatic interactions, and thus promote the hetero-aggregation between microalgae and MPs [24]. Hetero-aggregations were also observed by Huang et al. [25]. In order to further verify the secretion of EPS, the variations of organic matter composition in the synthetic piggery digestate were determined by 3D-EEM. Figure 5 showed the 3D-EEM images of synthetic wastewater from treatment of CK (a), PVC MPs (b), PP MPs (c), and PET MPs (d). In the emission wavelength (Em) from 220 nm to 540 nm and excitation wavelength (Ex) from 200 nm to 650 nm, the characteristics of electron emission spectra were divided into five regions [26]. They were named tyrosine-like aromatic protein (region I and region II) (Ex < 250/Em < 380 nm) [27], fulvic acid-like matter (region III) (Ex < 250/Em < 380 nm) [28], microbial metabolic byproduct-like matter (region IV) (Ex > 250/Em > 280 nm) and humic acid-like matter (region V) (Ex > 280 /Em > 380 nm) [27], respectively. Peaks enhanced in the region V after treatment by PVC and PET MPs, indicating the secretion of humic acid-like matter by microalgae. Peaks enhanced in the region II, III, IV, and V after treatment by PP MPs, indicating the secretion of tyrosine-like aromatic protein, fulvic acid-like matter, microalgae metabolic byproduct-like matter, and humic acid-like matter by microalgae. Microalgae can produce fluorescent substances during the growth process. There should be some kind of correlation between algae density and fluorescence intensity [29].

## 4. Conclusions

In this study, PVC, PP, and PET were selected to investigate the effect of MPs on the removal of nitrogen and phosphorus from synthetic piggery digestate by microalgae. The effects of MPs on the growth of microalgae and the removal of nutrient were dependent on the type of MPs and their particle size and content. PVC MPs particle size was observed to affect the growth of microalgae significantly, whereas the effects of PP and PET MPs on the growth of microalgae were not significant. The removal of NH_4_-N by microalgae was significantly affected by PVC and PP MPs with different particle sizes and contents. The removal of TP by microalgae was significantly affected by PVC, PP, and PET MPs with different particle sizes and contents. MPs increased the extracellular polymers (e.g., protein, fulinic acid, and humic acid) secreted by *Desmodesmus* sp. CHX1 thus influenced the removal of NH_4_-N and TP.

## Data Availability

The data presented in this study are available on request from the corresponding author.
